# Community case study: Community-driven strategies to build a socially inclusive rural county

**DOI:** 10.3389/fpubh.2025.1644090

**Published:** 2025-11-27

**Authors:** Elizabeth Ablah, Nadine Long, Christina M. Pacheco, Maddi Sutter, Shelby Bohnert, Sharon Fitzgerald Wolff, Sarah Finocchario-Kessler

**Affiliations:** 1 Department of Population Health, University of Kansas School of Medicine-Wichita, Wichita, KS, United States; 2 Sauerwein-Long Consulting, LLC, Andover, KS, United States; 3 Department of Family Medicine, University of Kansas Medical Center, Kansas City, KS, United States; 4 Mitchell County Local Health Equity Team, Beloit, KS, United States; 5 Mitchell County Regional Medical Foundation, Beloit, KS, United States; 6 Department of Population Health, University of Kansas Medical Center, Kansas City, KS, United States; 7 Department of Family Medicine, University of Kansas Medical Center, Kansas City, KS, United States

**Keywords:** social isolation, social inclusion, rural, participatory, community-driven

## Abstract

Mitchell County, Kansas is a rural county with a strong sense of local identity and a population committed to community well-being. Like many rural areas, however, residents experience structural challenges to social connection, including geographic isolation, demographic shifts, and limited access to inclusive services. These issues were further exacerbated by the COVID-19 pandemic. Through a federally funded initiative, Communities Organizing to Promote Equity (COPE), Mitchell County stakeholders developed community-driven strategies to address social isolation, with special consideration on how to include new immigrant residents. This case study outlines the intervention, centered on engagement and trust-building, cultural inclusion, employment access, and wrap-around supports; highlighting how other rural communities might replicate this work to build more welcoming, connected, and inclusive environments.

## Introduction / Nature of the problem

Social isolation, defined as “objectively having few social relationships, social roles, group memberships, and infrequent social interaction,” is a growing concern in the United States (1), (p. 321). The problem, identified by the U.S. Surgeon General in 2023 (2), is particularly acute in rural areas (3). Research indicates that geographic isolation, limited population density, limited access to fast internet connection speeds, and reduced access to shared public spaces contribute to social disconnection (4–8). In small rural communities, residents report that even long-term residents may experience social isolation (9). At the same time, newcomers experience added challenges due to limited job opportunities, lack of established social supports and networks, and the absence of familiar cultural anchors in their new environment (10).

These challenges were heightened by the COVID-19 pandemic, which disrupted traditional support networks and deepened health and economic disparities (11). In rural communities, new residents have historically experienced social exclusion, often met with skepticism from long-established residents until they integrate and become familiar (12). Opportunities for social integration in rural communities can be scarce due to fewer public venues and more rigid sociocultural norms that may not accommodate newcomers (13, 14).

Addressing social isolation in rural settings requires tailored, place-based strategies that account for each community’s demographics, history, and capacity or willingness for change (15). This paper describes how Mitchell County, Kansas, a rural community undergoing demographic changes, addressed social isolation and inclusivity through an innovative, community-led equity initiative.

## Context / Setting and population

Mitchell County, Kansas, located in North Central Kansas, covers 719 square miles, with a total population of 5,719, yielding a population density of 8.8 people per square mile (16). Most residents (93.3%) identify as Non-Hispanic White (16), which is consistent with the historical population of the community. However, in the last several years the county has experienced noticeable demographic changes due to the recruitment of immigrant workers for agricultural support, with a focus on swine production (17). These new residents, often arriving with spouses and children, experience social and economic inclusion barriers. The process of immigration inherently necessitates some degree of adaptation on the part of immigrants, as they navigate new cultural, social, and institutional landscapes (12, 18–20). Concurrently, host communities are presented with opportunities to evolve and adapt in response to the presence and contributions of immigrants. Such interactions can foster the development of vibrant, inclusive, and integrated rural communities, where mutual engagement and shared growth enhance social cohesion (21, 22). In response to the population changes, local leaders in Mitchell County have worked to shift the county’s reputation from one of insularity to one of intentional inclusion.

**Figure 1:**
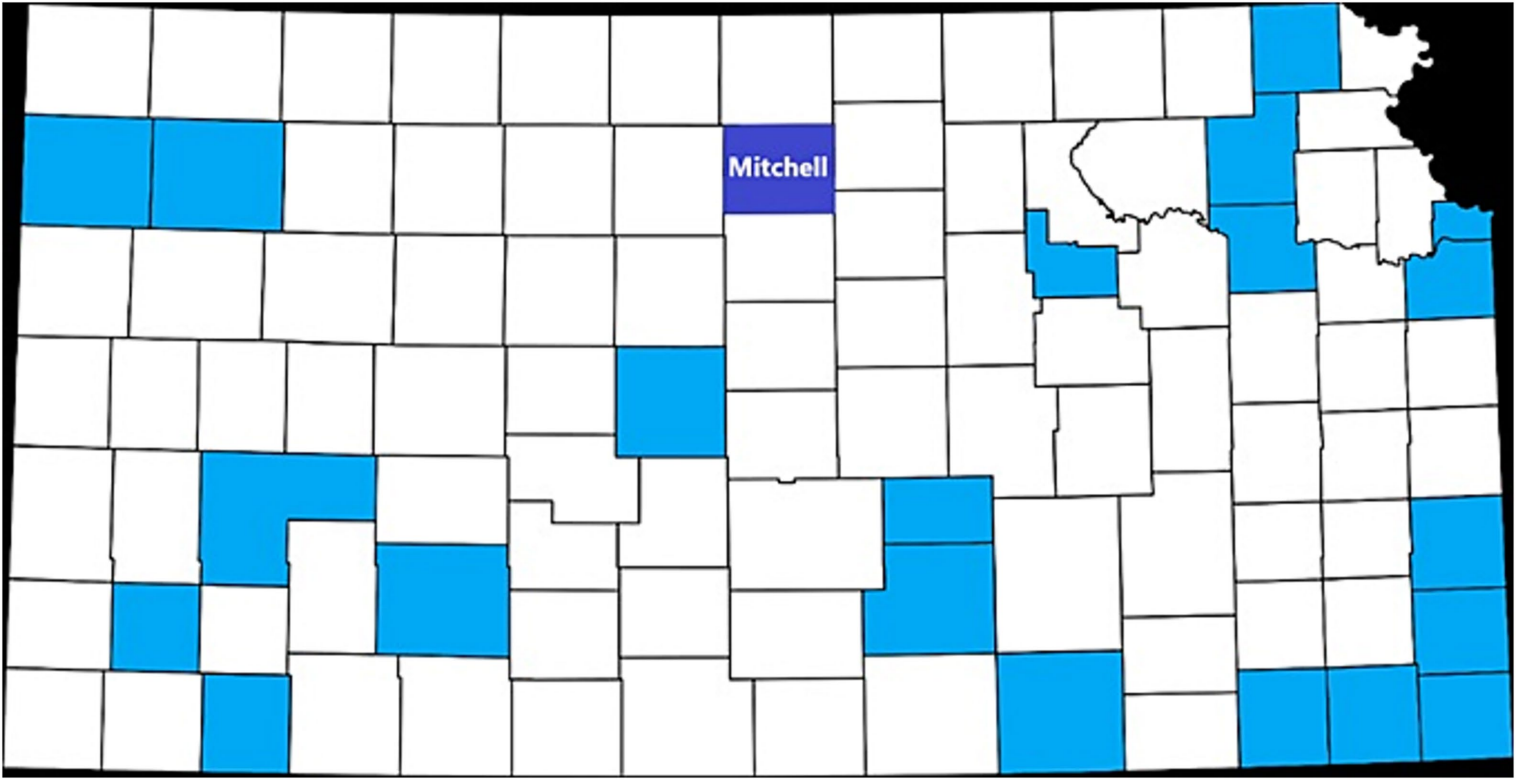
Map of Kansas counties participating in the COPE project, with Mitchell County highlighted in North Central Kansas.

In 2021, Mitchell County joined the Communities Organizing to Promote Equity (COPE) project (Figure 1), a Centers for Disease Control and Prevention-funded initiative implemented in 22 counties in Kansas by the University of Kansas Medical Center in partnership with the Kansas Department of Health and Environment. As described by Pacheco et al. (23), using a community-based participatory research framework, COPE supports counties in advancing health equity by addressing social determinants of health through community engagement and locally-driven interventions.

Each participating county formed a Local Health Equity Action Team (LHEAT), which brought together residents with lived experience with inequity, representatives from local organizations, and community health workers (CHWs) to identify and address barriers to health and well-being collaboratively. In Mitchell County, the LHEAT was initially led by staff from the Mitchell County Regional Medical Foundation, who supported the LHEAT’s vision of creating more opportunities for diverse community members to connect. The LHEAT was strategically assembled to ensure diverse and representative input, averaging five to seven consistent members at monthly meetings. It was comprised of: two to three CHWs, with slight fluctuations in number due to staff turnover over the course of the project; several community members who provided insights into the strengths and needs of the county; and representatives from local businesses, the school system, and local health department.

COPE counties pursued a wide range of equity-focused strategies to address systemic inequities. Although other communities incorporated elements of social cohesion and inclusion, the Mitchell County LHEAT uniquely prioritized inclusion as a long-term, intentional goal woven throughout all aspects of their equity work. This case study examines how the Mitchell County LHEAT responded to demographic changes and the problem of rising social isolation by promoting inclusion and a sense of belonging among all residents, with a particular focus on supporting immigrant families.

### Rationale for innovation

Early in their conversations about the well-being of their community members, the Mitchell County LHEAT identified a critical gap, the absence of visible inclusion and meaningful social cohesion for immigrant families newly settling in the area. In a candid discussion, LHEAT members reflected on a longstanding local culture described as *“you are not a real resident unless your family’s been here for seven generations,”* that contributed to a sense of exclusion for newcomers. Many immigrant residents, particularly spouses of agricultural workers, experienced social isolation and unemployment, often compounded by language barriers and a lack of familiarity with local systems and supports. The LHEAT wanted to transform Mitchell County into a place where all residents, regardless of their length of residency, felt a genuine sense of belonging.

## Key programmatic elements

The Mitchell County LHEAT implemented practical, community-led strategies to build a more socially inclusive and cohesive rural community. These interventions addressed interpersonal and structural barriers by prioritizing trust-building, promoting cultural exchanges, and offering supportive employment pathways. Although the activities employed could be adapted to fit any budget, the cultural inclusion program in Mitchell County cost approximately $22,000 over 18 months. This paid for space rental, the creation of a Cultural Inclusion Coordinator position that was hired by the hospital, language software licenses, and incentives for participants to complete the language lessons.

### Direct engagement and trust-building

Intervention strategies began with intentional relationship and trust building, particularly with newer immigrant residents. LHEAT members engaged in outreach by going door-to-door and spending time in informal social spaces, such as a popular local food truck. These efforts prioritized listening and relationship-building over service delivery, establishing credibility and rapport within the community. One LHEAT member dedicated several days each week over the course of a month to meet with residents, gather personal stories, and better understand residents’ experiences of isolation and exclusion. Over time, this LHEAT member was able to recruit one of the newly immigrated residents to join the LHEAT and contribute their perspective directly to the team, a testament to this LHEAT member’s dedication to developing meaningful connections with newer community members.

### Cultural inclusion program

To foster cross-cultural understanding and reduce social isolation, the LHEAT launched a monthly cultural inclusion program. Gatherings featured shared meals, unstructured social time, and bidirectional language learning activities, such as practicing greetings in Spanish and English. With more than 20 attendees per event, the dinners offered an opportunity for long-term residents and newcomers to connect in a welcoming, non-institutional setting. The structure encouraged mutual learning and normalized cultural exchange as part of community life.

### DEI policy and employment innovation

Recognizing the link between social inclusion and economic participation, the LHEAT was successful in engaging local institutions to operationalize diversity, equity, and inclusion (DEI) principles into their organizational culture (24). Due, in part, to their strong connection to the LHEAT, the local hospital led the way by developing a formal DEI statement as a framework for inclusive hiring and workplace practices. This effort informed similar strategies in the local school district (USD 273) and veterinary clinic. These organizations adapted employment pathways to accommodate non-English-speaking residents, including hiring spouses of agricultural workers, many of whom were previously unemployed, for school maintenance and support roles. These organizations take pride in the career pathways that have been created for these individuals as a direct outcome of these efforts. This approach filled critical labor shortages and fostered social and economic integration.

### Employer partnerships and wraparound support

To ensure the success of these employment efforts, the LHEAT actively cultivated partnerships with local employers. LHEAT volunteers translated application and onboarding materials, accompanied candidates to interviews, and advocated for culturally responsive hiring practices. COPE funds were used to provide tools such as Duolingo subscriptions, enabling residents to build Spanish and English language skills and further enhancing their integration into the workforce and broader community.

## Discussion / Implications and lessons learned

The Mitchell County intervention offers valuable lessons on how rural communities can foster inclusion through low-cost, community-centered strategies. Its success highlights the scalability of approaches that prioritize time, trust, and personal relationships over infrastructure or technology, an approach well-suited to resource-limited settings. This case study describes an approach that could be replicated in other rural communities experiencing social isolation or demographic change. By emphasizing coalition-building and culturally responsive engagement, the intervention has already demonstrated promising outcomes: within the first year, four immigrant spouse secured employment across three local organizations, immigrant families became more visibly involved in public events and community gatherings, and cross-sector collaborations (e.g., education, healthcare, local government) were strengthened.

Several key factors contributed to the success of this initiative. First, embedding inclusive values into organizational and community practices helped normalize conversations around equity and belonging. Second, authentic relationship-based outreach, anchored in face-to-face interactions and active listening, was foundational for building trust. CHWs and LHEAT members focused on community needs by meeting residents in informal community settings and listening rather than immediately offering services. Third, strategic engagement with the business community involved adapting diversity, equity, and inclusion (DEI) messaging. Recognizing that traditional DEI terminology could be alienating or misunderstood, the LHEAT adopted a values-based approach, using accessible language that emphasized dignity, respect, and compassion, values aligned with local cultural norms. Finally, continuous feedback from newer residents, especially immigrants, was integrated throughout planning and implementation. This iterative process ensured that interventions remained grounded in the lived experiences and aspirations of those most affected by social isolation and exclusion. Together, these elements demonstrate how inclusive, equity-focused strategies can take root and thrive in rural communities.

### Acknowledgment of any conceptual or methodological constraints

This study has several limitations. First, although the intervention was effective in Mitchell County, a small rural community in the Midwest, scaling to larger or more demographically diverse areas may require additional infrastructure and adaptation. The small population enabled a high-touch, relationship-driven approach that may not be directly transferable to other contexts. Furthermore, the LHEAT structure with CHWs and a Cultural Inclusion Coordinator created dedicated champions to achieve this change. Second, the findings are based on observational and anecdotal data collected throughout the project. A formal evaluation comparing pre- and post- intervention levels of social isolation was not conducted. Reflections from community members and project documentation suggest increased community participation and improved employment access for immigrant families, however, systematic data on health outcomes, employment stability, or long-term impacts are not yet available.

## Conclusion

Mitchell County’s experience demonstrates that small rural communities can meaningfully advance equity and foster inclusive cultures through intentional grassroots engagement, relationship-building, and community-driven interventions, reflecting a shared desire to act, even amid early uncertainty or fear about how to begin. By embedding inclusive practices into daily life and centering interventions on human connection, the county has created a replicable model for strengthening social cohesion in rural settings. This low-cost, relationship-centered approach requires a commitment to building trust, collaboration, and cultivation of a shared sense of belonging.

## Data Availability

The original contributions presented in the study are included in the article/supplementary material, further inquiries can be directed to the corresponding author.
